# Foundation of a Museum and Prizes, by Orfila

**Published:** 1853-05

**Authors:** 


*Foundation of a Museum and Prizes, by Orfila.*—We copy the following account of the foundation of the museum and prizes by the eminent and now venerable Orfila,* in the Dublin Medical Press. It will be read with interest, and may, perchance, suggest to some of the wealthy members of the profession in this country, the advantage which would accrue to American Medicine, as well as the honorable perpetuation of their names by similar acts of munificence.

"M. Orfila has just read the following letter before the Academy of Paris: "I do not wait, as is generally the rule, till death has removed me from

---

* Since this paragraph was penned the papers have announced the death of this distinguished member of the profession.

among you, to assign the sum of £4800 to different public establishments. I have two reasons for acting thus: first, because it is of some importance that the institutions to which I refer should as soon as possible reap the benefit of the donations which I am offering; and secondly, because I thought my presence would be of some use to overcome any difficulties which might arise during the carrying out of my plan; or perhaps in order to modify the latter, if the necessity of doing so were clearly demonstrated. I shall not attempt to enter into the reasons which have induced me to give the preference to certain institutions over others; it will be sufficient for me to state, that by giving £2400 to government for the completion of the museum which bears my name it is my intention to endow France with a scientific collection which will be unparalleled, and also to afford students in medicine a new proof of the sympathy and good-will with which I have always regarded them. I am also anxious to show them how grateful I feel for the very flattering attention they invaribly have given to my lectures for the last thirty-four years. I am anxious for this reason, that no misapprehension should exist regarding my motives, and have directed the following inscription to be placed over the principal entrance to the museum:—

"' *To Students in Medicine.*—I founded this museum, in 1845, for promoting medical studies, and solely to be useful to yourselves.—ORFILA.'

"I have thought it right to found a small annuity in favor of the keeper, who has always rightly attended to his duties. I also institute two prizes, one to be given by the Academy of Medicine (£80), the other by the School of Pharmacy, (£40), on subjects which have fixed my attention all through life. I have thus no other ambition but that of serving a science to which I have always remained faithful, and without allowing myself to be led astray by politics. I give to the Preparatory Schools of Bordeaux and Angea, £40 to the former, and £88 to the latter, to show how I approve of this kind of schools, which were organized upon a proposal of mine. To the Benevolent Medical Association of the Department of Seine, I give £16 a year, in proof of the high estimation in which I hold this society, which I am proud of having founded in 1833."

M. Orfila mentioned various other acts of kindness and benevolence of smaller importance, and received at the end of his discourse the hearty and unanimous applause of the members present. The Academy have decided that thanks should be tendered to M. Orfila by a deputation; and the medical press are calling a meeting for the same purpose."

